# Supplementary material for: Polyketide synthase gene clusters in the ericoid mycorrhizal fungus Oidiodendron maius: genome-wide prediction, expression profiling, and functional characterization of knockout mutants
Source: Front Plant Sci. 2026 Sep 16;17:1924430. doi: 10.3389/fpls.2026.1924430 (PMC13623903; doi:10.3389/fpls.2026.1924430)
Supplement: Supplementary file 1 [file SupplementaryFile1.zip › Supplementary Materials/Supplementary Folder 1.zip!/Oidma1_AssemblyScaffolds_Repeatmasked/knownclusterblast/cluster41/jgi.p_Oidma1_148727_mibig_hits.html]

| MIBiG Protein | Description | MIBiG Cluster | MiBiG Product | % ID | % Coverage | BLAST Score | E-value |
| --- | --- | --- | --- | --- | --- | --- | --- |
| ABB90277.1 | aldehyde\_dehydrogenase | BGC0001057 | Zearalenone\_biosynthetic\_gene\_cluster | 35 | 94.5147679325 | 237 | 3.4e-62 |
| CAQ52616.1 | benzaldehyde\_dehydrogenase | BGC0001066 | Kendomycin\_biosynthetic\_gene\_cluster | 35 | 90.5063291139 | 221 | 1.9e-57 |
| AFY23020.1 | betaine\_aldehyde\_dehydrogenase | BGC0000274 | Tetarimycin\_biosynthetic\_gene\_cluster | 34 | 90.5063291139 | 220 | 5.6e-57 |
| ABW71833.1 | putative\_aldehyde\_dehydrogease | BGC0000303 | Anthramycin\_biosynthetic\_gene\_cluster | 35 | 93.0379746835 | 214 | 3.1e-55 |
| AFT91402.1 | hypothetical\_protein | BGC0000338\_c2 | Echinocandin\_B\_biosynthetic\_gene\_cluster | 31 | 87.9746835443 | 211 | 2.6e-54 |
| ABC35115.1 | aldehyde\_dehydrogenase\_family\_protein | BGC0001102 | Malleilactone\_biosynthetic\_gene\_cluster | 33 | 90.2953586498 | 208 | 1.3e-53 |
| BAK64640.1 | putative\_aldehyde\_dehydrogenase | BGC0000135 | Reveromycin\_biosynthetic\_gene\_cluster | 32 | 93.0379746835 | 193 | 7.3e-49 |
| AAS47555.1 | putative\_aldehyde\_dehydrogenase | BGC0001108 | Pederin\_biosynthetic\_gene\_cluster | 31 | 94.5147679325 | 185 | 1.5e-46 |
| ABC36740.1 | aldehyde\_dehydrogenase | BGC0001120 | Burkholderic\_acid\_biosynthetic\_gene\_cluster | 31 | 95.9915611814 | 181 | 2.2e-45 |
| AAD32735.1 | MmcL | BGC0000915 | Mitomycin\_biosynthetic\_gene\_cluster | 30 | 91.9831223629 | 176 | 5.4e-44 |
| ABK32267.1 | AmbN | BGC0000014 | Ambruticin\_biosynthetic\_gene\_cluster | 30 | 91.9831223629 | 171 | 3e-42 |
| BAP16694.1 | aldehyde\_dehydrogenase | BGC0000376 | JBIR-34,\_JBIR-35\_biosynthetic\_gene\_cluster | 31 | 89.6624472574 | 170 | 5.1e-42 |
